# Cabinet of Curiosities: Venom Systems and Their Ecological Function in Mammals, with a Focus on Primates

**DOI:** 10.3390/toxins7072639

**Published:** 2015-07-17

**Authors:** Johanna E. Rode-Margono, K. Anne-Isola Nekaris

**Affiliations:** Nocturnal Primate Research Group, Oxford Brookes University, Headington, Oxford OX3 0BP, UK; E-Mail: eva.rode-2011@brookes.ac.uk

**Keywords:** *Nycticebus*, primates, Chiroptera, Eulipotyphla, Monotremata, venom delivery system, evolution

## Abstract

Venom delivery systems (VDS) are common in the animal kingdom, but rare amongst mammals. New definitions of venom allow us to reconsider its diversity amongst mammals by reviewing the VDS of Chiroptera, Eulipotyphla, Monotremata, and Primates. All orders use modified anterior dentition as the venom delivery apparatus, except Monotremata, which possesses a crural system. The venom gland in most taxa is a modified submaxillary salivary gland. In Primates, the saliva is activated when combined with brachial gland exudate. In Monotremata, the crural spur contains the venom duct. Venom functions include feeding, intraspecific competition, anti-predator defense and parasite defense. Including mammals in discussion of venom evolution could prove vital in our understanding protein functioning in mammals and provide a new avenue for biomedical and therapeutic applications and drug discovery.

## 1. Introduction

### 1.1. The Definition of Venom

Fry *et al.* [1] define venom as “a secretion, produced in a specialized tissue (generally encapsulated in a gland) in one animal and delivered into a target animal through the infliction of a wound (regardless how tiny it is). Venom must further contain molecules that disrupt normal physiological or biochemical processes so as to facilitate feeding or defense by/of the producing animal.” Fry *et al.* [1,2] caution against a traditional, anthropocentric view of toxicity, that acknowledges toxicity only if there are proofs of medical significance or effects on humans or laboratory animals. The authors prefer a definition based on biological functions that acknowledges for example that venom of specialized predators may be target-specific (e.g., birds, [3,4]) or some native prey can become resistant to predator venom, and thus do not show reaction. This contemporary definition of venom also recognizes animal clades that have not previously been regarded as venomous by traditional definitions, such as the haematophagus (blood feeding) fleas, ticks, leeches, and vampire bats [5], whose venom does not kill prey but facilitates feeding.

### 1.2. Venom in Mammals—An Unused Resource

Venom research can have biomedical and therapeutic applications and provide insights into venom evolution in biomedicine and pharma-therapeutics [6,7]. Due to the traditional definition of “venomous”, and the generally biased study towards well-known and more dangerous and dramatic species, the use of venom as a bio resource is still under-utilized [5,8]. Venom has evolved multiple times independently by convergent evolution in the animal kingdom, and occurs in centipedes, scorpions, spiders, several insect orders, cone snails, sea anemones, cephalopods, echinoderms, fish, toxicoferan reptiles and mammals [1,2,9,10,11]. Four lineages of venomous mammals are recognized yet their venom systems are comparatively little known [11,12]. Although traditional folklore and myths point towards the possibility that mammals could be venomous [12,13], the venomous members of this animal class have long been neglected by scientists. While new protein characterization and genomic techniques are available, laboratory tests are still restricted due to small quantities of available gland material, difficulties in maintaining some mammals in captivity, and the threatened status of several venomous mammal species [14]. Finally, many older studies have tested venom on laboratory animals instead of wild taxa [12]. Confirmed prey species or prey species from the habitat of the venomous species in question as may show the effect of venom better than the usual used mice, rats and rabbits. Prey-predator relations that shed light on the evolution of venomous mammals should be tested as well. Dufton [12] for instance points out that the order with the most venomous extant taxa Eulipotyphla (formerly known as Insectivora, see Section 3.2), shows an almost exclusive distribution with flightless birds, and suggests that birds should be explored in terms of venomous adaptations as well. Because mammals and especially primates are more closely related to humans, the study of venomous taxa in these taxonomic groups is especially interesting and important for the understanding of protein functioning, and applications in medicine and pharmacy.

### 1.3. Layout of this Review

In this review we summarize the current knowledge about venom systems and their functions in mammals, with more detail about primates. After we briefly discuss why venomous mammals are rare compared to other lineages in the animal kingdom, we consider the four different mammal lineages with confirmed venomous species. For each lineage we include aspects describing the “venom system”—the venom delivery apparatus, the venom gland and the secreted toxins [2]—and the suggested ecological functions of the venom. The venom delivery apparatus, the venom gland(s) including the connecting ducts and possible muscles involved in the delivery of the venom are referred to as the venom delivery system (VDS). In the animal kingdom many different VDSs have evolved to facilitate the delivery of venom into the target animal. The venom delivery apparatus can consist of a wide variety of fangs, or modified teeth, spines, spurs, stingers, pincers, sprays, and others [1,8,10,15,16].

## 2. Why Is Venom Use in Mammals Rare?

The reason why venom systems are so rare in extant mammals, while they are so manifold in other animal groups, and whether or not venom systems were present in early mammals, remains speculative. Folinsbee *et al.* [17] argue that the sophisticated mammalian masticatory apparatus led to a wide range of different feeding strategies making the use of venom redundant. Indeed, while many mammal orders are mainly herbivorous (e.g., Artiodactyla, Rodentia) or insectivorous with usually small prey relative to the predator’s body mass (e.g., Chiroptera), carnivorous species are mostly large and able to overcome their prey by their strength [18]. The earliest eutherian mammals developed during the late Cretaceous (66–144 Mya) [19] and had dentition and skeletons similar to extant shrews and hedgehogs [12]. Thus this clade forms a basal group for extant mammals. Dufton [12] argues that venom was more widespread in this ancestral group, as animals were small and imperfectly homothermous (warm-blooded) with a selective pressure of high foraging efficiency, with the use of venom giving them a selective advantage. The diverse geographic locations of present-day venomous Eulipotyphla (*Neomys* spp.: Europe, Asia; *Blarina* sp.: North America; *Solenodon* spp.: Greater Antilles, Caribbean) would further support this view. The fossil record may support the view that venom was more widespread in early mammals. Reconstruction of soft tissue structure and function from bones and teeth is difficult [20]. Two studies claimed to discover venomous extinct mammals from the Pleistocene and late Paleocene (*Bisonalveus browni*, *Beremendia fissidens* and an indeterminate soricine) based on grooves running along their teeth that potentially could aid in venom delivery [21,22]. Inferences were criticized by Folinsbee *et al.* [17] and Orr *et al.* [20] who argue that traits should be present in analogous extant taxa and the association of the trait and the function should be present in all taxa. Orr *et al.* [20] used a comparative approach that showed that several non-venomous mammals have grooved teeth probably functioning as structural support of teeth in fights (e.g., in some primates, hippos or suiforms) and most of the venomous mammals except for solenodons have non-grooved teeth. As Cuenca-Bescos and Rofes [22] found fossa (small holes) within the mandibular symphysis improving efficient toxic saliva transmission, their conclusion of having found a venomous extinct mammal is more credible [17].

## 3. The Venom System and Its Functions in Mammals

### 3.1. Chiroptera

**Species.** The blood-sucking vampire bats (Table 1) comprise three genera with one species each, together forming the subfamily Desmodotinae (family Phyllostomidae—New World leaf-nosed bats) and exclusively occurring in South and Central America.

**Figure 1:**
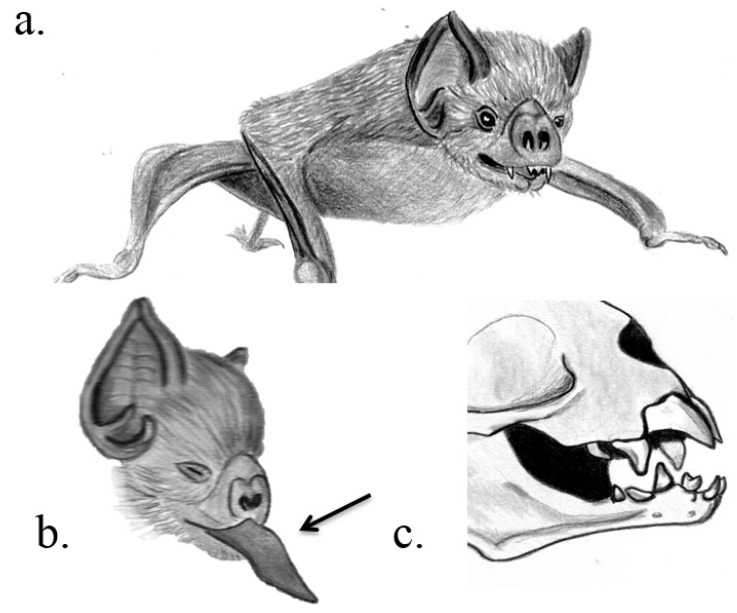
Venom system of vampire bats. Common vampire bat *Desmodus rotundus* (**a**); with specialized tongue as indicated by the arrow (**b**); and teeth (**c**) Illustrations: Kathleen Reinhardt.

**Venom delivery system.** Vampire bats feed on blood. The VDS consists of modified large and sharp incisors that inflict crater-like wounds to the prey animal, submaxillary venom glands and a tongue that darts in and out of the wound to deliver venom from its sides [23] (Figure 1). The bat sucks the blood up through two ducts on either side of the tongue [23]. Target animals are usually cattle, horses, goats, pigs, sheep, or birds. Bats prefer sleeping prey and they approach them carefully. Their bite is described as painless.

**Venom composition.** The venom of vampire bats possesses strong anticoagulant and proteolytic activity that delays blood clotting for several hours [11]. Two venom components that have been studied are draculin, an anticoagulant [24], and plasminogen activators or Desmokinase (*Desmodus rotundus* salivary plasminogen activator—DSPA), which dissolved fibrin clots to allow a continuous blood flow [25,26] as well as several previously unknown scaffolds of proteins (Low *et al.*, 2013).

**Table 1:** Venomous mammals and their venom systems. VDA = venom delivery apparatus, PC = prey capture, IC = intraspecific competition, PD = predator defense.

Order, Family	English Name	Scientific Name	VDA	Venom Gland Position	Ecological Function	References
Chiroptera, Phyllostomidae	Hairy-legged vampire bat, white-winged vampire bat, common vampire bat	*Diphylla ecaudata*, *Diaemus youngi*, *Desmodus rotundus*	Razor-like upper and lower incisors	Principal submaxillary gland	Facilitation of feeding	Low *et al.* 2013
Soricomorpha, Soricidae	American short-tailed Shrew, European water shrew, Mediterranean water shrew	*Blarina brevicauda*, *Neomys fodiens*, *N. anomalus*	Sharp and large incisors and canines	Significantly enlarged and granular submaxillary salivary glands	Unclear Possible: PC, prey immobilising agent, digestive aid	Tomasi *et al.* 1978, Martin 1981, Lopez-Jurado & Mateo 1996, Kita *et al.* 2004, Dufton 1992
Soricomorpha, Solenodontidae	Hispaniolan solenodon, Cuban solenodon	*Solenodon paradoxus*, *S. cubanus*	Enlarged and modified lower second incisors with almost tube-like deep groove	Submaxillary glands near base of the tubular lower second incisors	Unclear Possible: PC, IC	Orr 2007, Folinsbee *et al.* 2007
Monotremata, Ornithorhynchidae	Platypus	*Ornithorhynchus anatinus*	“Crural system”: Hollow keratinised spurs on hindlegs connected by a duct to the venom gland	“Crural glands”: Specialised venom glands in thigh area	IC (sexual competition during mating season), PD	Temple-Smith 1973, Whittington & Belov 2007, Krause 2009, Grant & Temple-Smith 1998
Primates, Lorisidae	Slow and pygmy lorises	*Nycticebus* spp.	Needle-like toothcomb (incisors and canines of lower jaw)	“Brachial gland”: Venom gland on the ventral side of the upper arm, submaxillary saliva gland	Unclear Possible: PC, PD, IC and/or ectoparasite defence	Nekaris *et al.* 2013, Hagey *et al.* 2007, Krane *et al.* 2003, Alterman 1995

**Ecological functions** Vampire bats are highly specialized for a hematophagus lifestyle with sensory ability to locate prey; the position of capillaries, and strong limbs aid in approaching prey on the ground [23,27]. Their venom system developed to serve the ecological function of facilitating feeding. A normal haemostatic (stopping blood flow) response after a wound is inflicted would be the fast production of a fibrin clot that prevents further blood loss. Target animals normally do not die, thus the relationship to the target animal is more that of a parasite that ensures the continuous survival of the host animal [28]. Prey animals develop an immune response with resistance to anticoagulants, with regularly exposed animals showing shorter blood-clotting and bleeding times [28].

### 3.2. Eulipotyphla

**Species.** Formerly known as Insectivora, this Order includes the highest number of recognized venomous mammal species, including three species of shrews and two species of solenodons (Table 1). The shrew species (family Soricidae—shrews) belong to the subfamily Soricinae (red-toothed shrews) occur in western North America (*Blarina brevicauda*), Europe (*Neomys anomalus* and *N. fodiens*) and parts of Asia (*N. fodiens*). The two species of the family Sonenodontidae occur on Cuba (*Solenodon cubanus*) and the Dominican Republic and Haiti (*S. paradoxus*). There is still suspicion whether the Canarian shrew *Crocidura canariensis* [29], the American shrew *Sorex cinereus*, and the European mole *Talpa europaea*, family Talpidae, are venomous [11,29]. Lopez-Jurado and Mateo [29] showed that Canarian shrews can paralyze lizards with their bites. Moles are known to cache paralyzed worms in their burrows, similar to shrews, and have large and granular maxillary glands [12]. These species have not yet been tested for venom [11].

**Figure 2:**
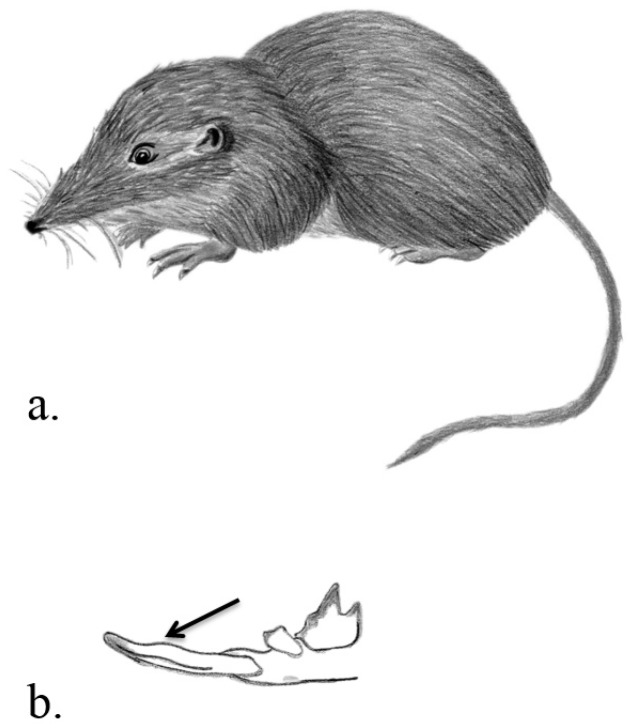
European water shrew *Neomys fodiens* (**a**); with concave incisor surfaces (as indicated by the arrow) that help with flow and injection of venom (**b**). Illustrations: Kathleen Reinhardt.

**Figure 3:**
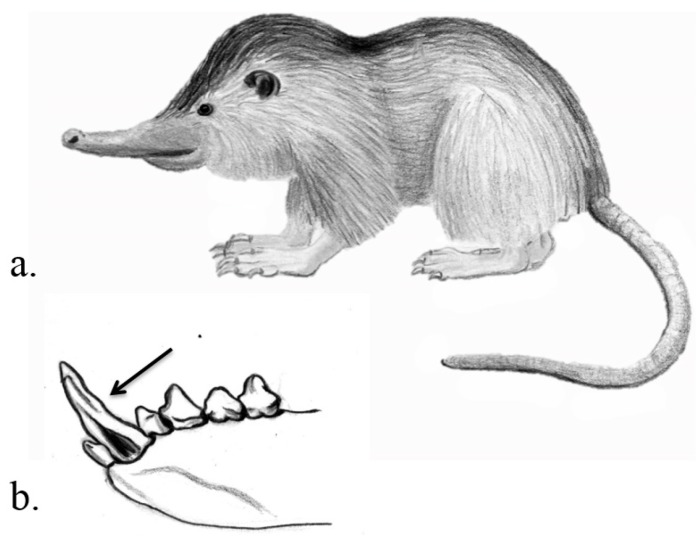
Venom system of solenodons. Hispaniolan solenodon *Solenodon paradoxus* (**a**); with deeply grooved lower canines (as indicated by the arrow) that aid in flow and injection of venom (**b**). Illustrations: Kathleen Reinhardt.

**Venom delivery apparatus.** In all species the VDS involves enlarged and granular submaxillary glands where toxic saliva is produced. The animals inject the venom with their teeth. Shrews have sharp and large incisors and canines as typical for insectivores. The teeth are ungrooved but incisors have concave inner surfaces [17] (Figure 2). Solenodons in contrast possess lower enlarged canines that are deeply grooved [17] (Figure 3). In shrews the glands are ducted towards the front of the lower jaw [12], and in solenodons pockets hold the venom glands inferior to the base of the teeth [17].

**Venom composition.** One of the toxic components of the venom of the American short-tailed shrew is blarina toxin (BLTX) that can be extracted from the sublingual and submaxillary glands [30]. This neurotoxic protein is responsible for the main effects on tested target animals (mice, rabbits, cats, insects) such as general depression, breathing disturbance, paralysis and convulsions, especially if injected intravenously [12,30,31,32]. Similar effects have been observed for *Neomys* spp. and solenodon venom [33,34], but the toxin has not been purified yet. Another kallikrein-like protease, Blarinasin, has been purified from the salivary glands of *Blarina brevicauda* and shows a high similarity to BLTX [35]. It has not revealed toxic effects to laboratory mice [35] but may add to the toxic effect of shrew saliva on other taxa.

**Ecological functions.** There are still debates about the ecological function of venom in shrews and solenodons [11]. Due to their small size and high metabolism, shrews need a constant food supply and consume more than their body weight within 24 h [12]. They are known to immobilize and cache their prey (especially earthworms, insects, snails, small mammals) for later consumption. This hoarding of live but paralyzed prey may especially be advantageous in cold seasons with infrequent and lower quantity or quality food supply [32,36]. Others state that the possession of venom would enable shrews to overcome larger prey by adding to their power to weight ratio [12,37]. Although shrews are very fast and fierce hunters, venomous bites in the occipital region of the head of fishes, frogs, mice and voles may help to save energy when overcoming prey [12]. The proportion of large vertebrate prey for instance in the diet of *Nyomis fodiens* is relatively small and the main bulk consists of small vertebrates [38,39]. Although shrews can kill mice and frogs in captivity [31,40], Harberl [39] points out that shrews have not been reported killing rodents in the wild, but that they feed on rodent carcasses. Wolk [38] reports a seasonal preference for frogs in the winter, but notes that the amphibians were relatively immobile due to temperature. It is possible that shrew venom has mainly evolved as an invertebrate immobilizing agent instead of overcoming large prey [32]. Due to the relatively high food intake in shrews, Dufton [12] also discussed a possible digestive aid by venom. It is not yet clear if the tooth canals in solenodons have specifically evolved to facilitate venom injection or if they merely serve structural stability [17]. Finally, Rabb [34] observed that Hispaniolan solenodons kept together in enclosures had high death rates despite the only visible wounds being bite marks by conspecifics on the feet. Thus, a function as a weapon in intraspecific competition cannot be ruled out for solenodons.

### 3.3. Monotremata

**Species.** The platypus (*Ornithorhynchus anatinus*), the only extant species in the family Ornithorhynchidae, lives in fresh water rivers and streams on the east coast of Australia [41]. Members of the only other family of monotremes (family Tachyglossidae—echidnas), the related long-beaked echidnas (*Zaglossus* sp.), have spurs (raised pointed regions on the ankles made of cartilage) like the platypus (see below), but they cannot be erected [7]. A milky substance is secreted in the breeding season, which may act as communication [7]. The transcriptome of the echidna crural gland revealed few similarities in expressed genes, and although a few toxins could be detected, they showed low expression in the echidna [7]. As it has been shown for the reptile clade Toxicofera, venom system can be secondarily lost in evolution [2,42]; e.g., if snakes shift their prey capture technique to constriction or their prey type to defenseless prey such as eggs, worms or snails [2,8,42]. Thus, it is possible that the echidna used to be venomous but lost it in the course of its evolution [7].

**Figure 4:**
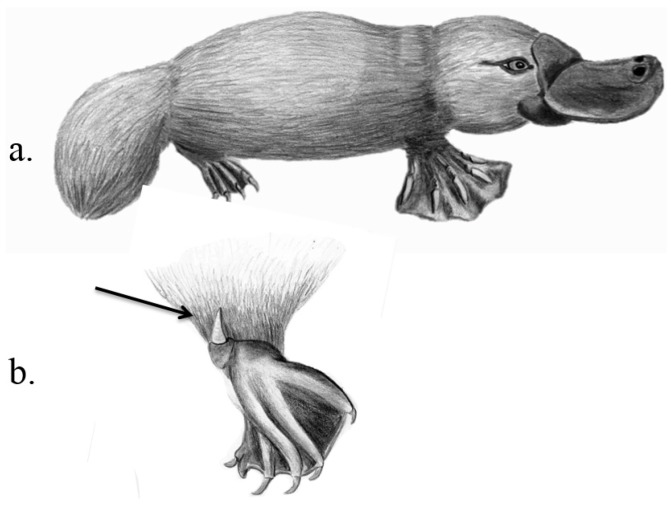
Venom system of the platypus. Platypus *Ornithorhynchus anatinus* (**a**); with crural spur as indicated by the arrow (**b**). Illustrations: Kathleen Reinhardt.

**Venom delivery apparatus.** In adults, the VDS is only present in males that possess hollow keratinized spurs on their hind legs that are connected to the venom-producing crural glands (sac-like alveolar glands in the upper thighs) (Table 1; Figure 4). Spurs and glands together are called the crural system. The spurs can be erected with the help of strong muscles and small articulating bones, and driven into the target animal [43,44]. To attack, animals wrap their hind legs around the target animal, drive their spurs into it and venom is injected [45]. Spurs and muscles are so strong that it is difficult for a victim to expel the attacking platypus. Both sexes are born with spurs, but females lose them during ontogeny [43].

**Venom composition.** While Whittington *et al.* [46,47] used genome sequence and next-generation transcriptome sequencing to identify a range of putative toxins in the venom of platypus, Wong *et al.* [6] used proteomic analysis and comparisons of transcriptomes between seasons and identified ten proteins in the platypus venom: Nerve growth factor, C-type natriuretic peptides, venom defensin-like peptides antimicrobials, amide oxidase, serpin protease inhibitor, proteins associated with the mammalian stress response pathway, cytokines, and other immune molecules. Early tests on rabbits revealed the effects edema, hypotension, respiratory problems, intravascular coagulation and death [48,49], while envenomated people describe intensive pain and swellings lasting for weeks or even months with no effect of first aid pain killers such as morphine [45].

**Ecological functions.** It is believed that the venom system has its function in sexual competition for females [11,44], as venom glands are only active in the mating season [50] and show seasonally distinct gene expression profiles [6]. Males generally avoid each other and become highly territorial and aggressive during the mating season [50]. Platypus venom may also have defensive functions. When the platypus was hunted for its fur, envenomation of people and (hunting) dogs occurred [43]. In contrast to humans, dogs have been killed by the platypus’ venom [43].

### 3.4. Primates

**Species.** Eight species of slow lorises (*Nycticebus* spp.) are currently recognized in the family Lorisidae, distributed from NE India to the Philippines and Indonesia, and are the only primates that are known to be venomous (Table 1). So far only three species have been tested for venom (*N. bengalensis*, *N. coucang* and *N. pygmaeus*) but observations suggest that the other species are equally venomous.

**Figure 5:**
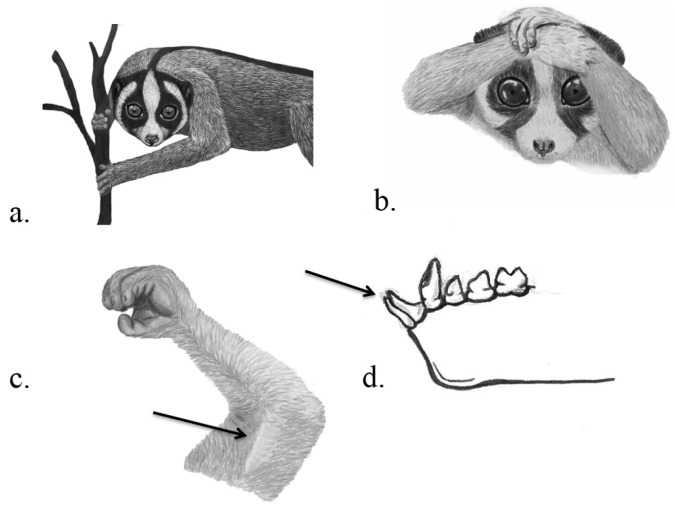
Venom system of slow lorises. Javan slow loris *Nycticebus javanicus* showing warning coloration of face (**a**); Javan slow loris displaying defense position (**b**); brachial gland as indicated by the arrow (**c**); tooth comb as indicated by the arrow (**d**); Illustrations: Kathleen Reinhardt.

**Venom delivery apparatus.** The VDS consists of the brachial gland that is located in a relatively hair-free, slightly raised area in the flexor region of the upper arm [51], and the needle-like toothcomb, a compression of the anterior teeth of the jaw comprising the canines and incisors (Figure 5). When threatened, the slow loris can “charge” its VDA by raising its arms over the head to combine brachial gland exudate (BGE) with saliva [51]. The powerful and sharp toothcomb is usually believed to aid in feeding and grooming but has been shown to enable venom to travel upwards to the tip of the tooth by capillary forces [52]. Wounds inflicted from slow loris bites are very painful, slow healing, can cause swelling, local loss of feeling, fester, and leave scarring and loss of fur in conspecifics [53,54,55]. In other slow lorises, bite wounds appear as a black scab overlying green-coloured slough; in such wounds, necrosis radiates from a central position, assumed to be the entry point of the tooth and venom [56]. Reactions in humans range from little effect to severe anaphylactic shock, including hypotension, tachycardia, backache, poor organ perfusion and peripheral shut down that may even lead to death [53,57]. To other animals, slow loris venom can also be lethal. Pramaswari *et al.* [56] recorded 40 venomous bites in 25 slow loris individuals within two weeks of arriving at a rescue centre in Java, resulting in the death of four individuals. Alterman [52] injected two different extracts of BGE (with formic acid and methylene chloride) into mice. The extracts of only 2 of 10 and 4 of 7 slow lorises were lethal to mice. When he incubated BGE with saliva, all mice died. He suggested that the toxic proteins in the BGE must be activated by enzymes in the saliva. Grow *et al.* [58] showed that BGE and saliva combined was also lethal to arthropods. This two-stage venom is unique in the animal kingdom.

**Venom composition.** Composition of slow loris venom is currently only known from captive-born animals. Krane *et al.* [59] extracted BGE from a single animal, probably Bengal slow loris (*N.* *bengalensis*), and used high performance liquid chromatography to identify organic compounds in the venom sample. They found that the BGE protein had a high sequence similarity to the cat allergen Fel-d1 and suggested that this similarity to an allergen might explain the variable reactions to slow loris bites in humans. Hagey *et al.* [51] further examined this major component and identified it as a new member of the secretoglobin family. This heterodimeric protein with 17.6 kDa has an α-chain and a β-chain that have high sequence similarity with the two chains of Fel1d. All three slow lorises species tested (greater slow loris *N. coucang*, *N. bengalensis*, pygmy slow loris *N. pygmaeus*) have two protein isoforms [51,59]. They also found that the BGE is unique and complex oil and contains more than 68 (*N. bengalensis*) and 200 (*N. pygmaeus*) volatile and semi-volatile components.

**Ecological functions.** Although several non-exclusive hypotheses have been proposed to explain the ecological functions of slow loris venom [51,52,58,60,61], the main purpose of the venom still remains unresolved. Variations in venom composition in relation to different variables, such as sex and reproductive status of the slow loris, season or diet, could not be tested yet due to difficulties in exporting a meaningful amount of samples from range countries. So far only behavioral observations of wild animals and behavioral experiments with captive animals, the latter having to comply with welfare standards, could be used to shed light on the most likely ecological functions. Although some functions seem to be more likely than others, not enough work has been done yet to confirm a leading theory.

#### 3.4.1. Intraspecific Competition

Currently among the most likely theories to explain the function of venom in slow lorises is intraspecific competition. Only a few species are reported to use venom in intraspecific competition; in mammals this was only suggested for the platypus (see Section 3.3). The second gnathopods or ghost or skeleton shrimps (*Caprella* spp., order Amphipoda, family Caprellidae) are armed with a so-called poison tooth that is connected to a venom-producing gland [62]. Male second gnathopods have larger teeth, which they use in often-fatal combats with sexual competitors [62]. Cone snails (superfamily Conidea) use their extendible proboscis and a needle-like radular tooth that are connected to an esophageal venom gland to prey on worms, molluscs or fish [63]. Olivera *et al.* [63] report that cone snails not only catch prey with the help of venom, but also use it against potential predators and in intra- and interspecific competitive interactions. Intraspecific slow loris bite wounds are common in the wild and captivity with severe health consequences such as necrosis, septicaemia, lung edema, and cellulitis, which are chronically non-healing and often lead to death [64,65,66]. The anaphylactic shock in humans reported by Wilde [53] occurred after the owner attempted to separate two fighting lorises. Although agonistic encounters are infrequently observed in the wild, males compete intensively for females during mating, same-sex conflicts occur at territorial boundaries, and wound rate is high in caught animals [67,68]. Similar to the venomous platypus, loris venom is used in sexual competition [11,44], and male slow lorises anoint themselves before and during agonistic encounters by grooming their brachial gland and then their own fur [60]. Continued detailed observations of wild and captive slow lorises in competitive situations, and the analysis of variations in venom composition in relation to respective variables such as sex or reproductive status may further confirm this hypothesis.

#### 3.4.2. Predator Defense

Predation would seem to be a driving force in the selection of venom, yet evidence that slow lorises use their venom against predators is mixed. A weapon such as venom aiding in defense would be advantageous against predators. Although slow lorises can walk and climb relatively fast, they cannot agilely leap away from potential predators [69]. In the typical defense position where they raise their arms and interlock them above the head (Figure 5), slow lorises smear the strong smelling venom to the head and neck. It was suggested that slow lorises use venom directly against predators by biting and injecting the venom [52], or indirectly by warning conspecifics through the smell of increased BGE secretion, by deterring predators with olfactory cues in the slow loris’ gland exudates (Muellerian mimicry) [51] or by anointing to conceal adults and their offspring (olfactory crypsis) [52]. Slow loris infants are “parked”, e.g., left alone in the vegetation when the mother is foraging actively during the night [70]. They can be parked from the day they are born (although normally mothers carry them for the first six weeks), with the duration of time being parked gradually increasing [71], leaving them completely unprotected. Although Nekaris *et al.* [60] only observed one event where a mother anointed a parked infant in 18 months field observation, anointment with a defensive smell would be beneficial during this vulnerable state of the young. If the venom has a repellent effect, this could be due to a smell advertising unpalatability, a camouflaging smell or a chemical warning signal of the actual venom. Many mammal species use scent as a repellent, and chew plant material with secondary metabolites and rub it on their fur [72,73], or ingest material and accumulate toxins in their fur or feathers to make themselves unpalatable (*Pitohui* *Ornorectes*: [74]; poison dart frogs Dendrobatidae: [75]; rough-skinned newt *Taricha granulosa*: [76]). Ground squirrels (*Spermophilus beecheyi*, *S. variegata*) are reported to chew rattlesnake skins (*Crotalus* spp.) to deter these known predators [73]. Field observations support the notion that young lorises may be more “toxic” than adults. An 80 kg adult man bitten by a ~0.4 kg juvenile *N. kayan* had a severe anaphylactic reaction [57]. As opposed to variable reactions in adults, all immature slow lorises that were captured by the authors in a study on Javan slow lorises (*N. javanicus*) clearly secreted venom and showed more aggressive reactions, as well as immediately assumed defensive postures (JRM and AN, unpub. data). Casewell *et al.* [9] doubt the adaptiveness of venom as a predator defense strategy if predator encounters are relatively rare and predators diverse. The prediction that the venom would directly repel predators seems, however, to at least hold true for olfactory-oriented predator species. In behavioral experiments, the mix of BGE and saliva effectively repelled cats (leopard *Panthera pardis*, tiger *P. tigris*, clouded leopard *Neofelis nebulosa*), sun bears (*Helarctos malayanus*) and civets (common palm civet *Paradoxurus hemaphroditus*, binturong *Arctictis binturong*), but not visually-oriented Bornean orang-utans *Pongo pygmaeus* [52,60]. The fact that Javan slow lorises seem to be unconcerned by common palm civets and leopard cats (*Prionailurus bengalensis*) was confirmed in the field where adult and young slow lorises move in close distance of less than 5 m of the potential predators [77]. Visually-oriented predators, even genera known to consume wild slow lorises [78], showed little to no reaction to slow loris venom. Bornean orang-utans actually eagerly consumed swabs containing loris venom [60]. *Spizaetus* and *Spilornis* eagles also consumed swabs containing loris venom, but did show behaviors indicating irritation, especially perch rubbing; these behaviors however were not significant [61].

#### 3.4.3. (Ecto-) Parasite Defense

A possible side effect of venom production by slow lorises is its use in ectoparasite defense. Ecotparasites negatively affect success in reproduction and survival [79]. Many species thus reduce parasite load with the help of secondary metabolites [80]. Several bird and mammal species including primates are known for anting (letting ants walk over their fur or plumage) or anoint themselves with other plants and animals (e.g., millipedes, lime fruits *Citrus*, leaves and stems of vines, resins) that have bioactive compounds reviewed in [80,81,82]. Many species first chew plant parts to release the active compounds and mix them with saliva for easier application. These treatments are believed to have an anti-parasitic effect [81]. Several bird species are known to add fresh leaves with insecticidal and antibacterial properties into their nests [82]. While in gregarious primates grooming serves to reduce parasite load [83], species that have a solitary or dispersed social organization lack this service by conspecifics and are not able to clean fur in inaccessible body regions [84]. This is especially the case when species like slow lorises go into solitary torpor or park their young during active foraging periods [68]. The venom of slow lorises may have a similar repellent effect on ectoparasites [60]. Prevalence and intensity of ecto-parasite infestation among Lorisidae is extremely low compared to other primates. While eight of nine wild studies of six taxa revealed no or few ecto-parasites (slender lorises *Loris tardigradus*, *L. lydekkerianus lydekkerianus*, *L. l. nordicus*, Bengal slow loris, Javan slow loris, pygmy slow loris), only one study of greater slow loris conducted during the wet season found a small amount of ticks in all animals [60,85]. All twelve leeches used in a preliminary test died upon coming into contact with BGE combined with saliva [60]. Grow *et al.* [58] tested the effect of BGE on arthropods and found 78% of arachnids died within one hour after the mixture of BGE and saliva was applied. Ticks are members of the arachnid order. As ectoparasite infection varies across season [86] a co-varying toxicity of venom may indicate that slow lorises use venom for ectoparasite avoidance and defense.

#### 3.4.4. Prey Capture

Evidence is very weak that slow lorises use their venom to acquire prey. In Alterman’s [52] experiments BGE combined with saliva was lethal to mice. Yet, although slow lorises feed on large insects and small vertebrates (birds, frogs, lizards, mice, bats, tarsiers), in contrast to shrews, prey is still relatively small compared to the predator’s body size. Slow lorises catch and consume prey rapidly and effectively, and there is an indication neither of paralysis in prey nor of caching behavior in slow lorises [60]. Captive behavioral experiments report that slow lorises are highly capable in killing prey, and do not seem to use venom for killing [87]. Experiments that involve the application of BGE and saliva on arthropods showed that in 84% of the trials maggots (a common food of slow lorises) were initially impaired but only 42% died after one hour [58].

### 3.5. Arguably Venomous Species

The definition of Fry *et al.* [1] mentions three aspects of venom: the production in a specialized gland, the delivery of the venom through the infliction of a wound, and the subsequent disruption normal physiological or biochemical processes. Two species, the European hedgehog *Erinaceus europaeus* and the African crested rat *Lophiomys imhausi* have been suggested to venomous, but “borrow” their venom from other organisms [88,89]. As they do not comply with the full definition due to a lack of a venom gland, they may hold a special position between truly venomous and poisonous animals. Further research may reveal that the species’ saliva may augment the borrowed toxins, thus playing an active role in processing toxins, as suggested for the unusual large salivary glands of African crested rats [89]. European hedgehogs are thought to anoint their spines with toxic saliva mixed with toad (*Bufo*) as a predator defense strategy, but tests could not yet verify toxic substances [88]. A similar behavior was described for the African crested rat [89]. Animals chew roots and bark of *Acokanthera schimperi* (Apocynaceae) trees and apply the saliva onto their VDS that consists of specialized lateral-line hairs [89]. The sponge-like structure of the hairs allows the saturation with toxic liquid aided by capillary forces [89]. Upon being attacked, the animal parts the long, covering hair with specialized muscles so that the toxin-loaded hair is exposed [89]. Venom is likely to be “ouabain” that can be extracted from the *Acokanthera* tree [90] and is traditionally used in Africa for elephant hunting [91]. The toxin seems to be effective in deterring predators like domestic dogs [89]. The mucous membranes of dogs that try to bite an African crested rat come in contact with the rat’s toxin-loaded hairs that can cause lack of coordination, mouth frothing and distress, but may even lead to collapse and death [89]. Physiological effects include heart failure, defective blood-clotting and generalized internal bleeding [89]. White blood cells with toxic granules were found. In human medicine, ouabain can be used to treat hypotension and cardiac arrhythmias [92]. An endogenous oubain has been isolated and identified from mammalian tissues including human plasma, and likely plays a role in hypertension and the pathogenesis of heart and renal failure [93]. Venom in African crested rats seems to serve a predator defense function [89].

## 4. Looking Forward

Within mammals, venomous systems appear to have evolved multiple times, with the ecological factors driving selection of such systems ranging from foraging, to predation, to mating systems. Our research on Primates shows that venom may have multiple functions within a single animal lineage. Using venom for multiple purposes does not only occur in mammals; while the most common ecological function of venom in the animal kingdom is prey acquisition [10], venom in some species has initially or primarily evolved for one purpose, but gained usefulness for another, secondary function [10]. Spitting cobras for instance are one of the rarer reptile species that use venom for both defensive purposes and prey capture [94]. New definitions of venom allow for the first time for studies of mammalian venomous systems to be explored in more detail and for new venomous taxa to be sought. While species such as monitor lizards (*Varanus* spp.) or vampire bats have not been regarded as truly venomous until recently, studying the effect on natural prey instead of laboratory animals, and the view that venom does not necessarily need to kill prey have shown that they are venomous. Acceptance of these definitions will further broaden the spectrum of venomous animals, and helps to explain peculiar adaptations in numerous taxa, their evolution and their natural history [94]. Comparisons to non-venomous taxa that are closely related to venomous taxa can give us insight into the evolution and secondary loss of venom [2,42,44].

Advances in genomic techniques, and proteomic and biochemical analyses helps to identify new toxins, shed light on their evolution, and answer questions like when in evolutionary history venom genes have been recruited, got retained and lost in mammals and other taxa [95]. Using the fact that the composition of venom can vary within one species and even individuals [94,95,96] may further help us to study venoms. Recent research on platypus venom for instance has used the completely sequenced genome in combination with next-generation sequencing of a gland transcriptome during the passive (non-venomous) and active (venomous) season to identify new toxins including five that are only known from platypus [95] and to reveal that not only gene duplication, but also mutations in regulatory or coding regions and alternate splicing [6].

Including mammals in discussion of venom evolution could prove vital in our understanding protein functioning in mammals and provide a new avenue for biomedical and therapeutic applications and drug discovery [97]. Due to our closer relatedness to mammals and primates in particular, more scientific attention to mammal venoms may imply a higher chance of finding applicable findings in this area.
